# New Appliances and Things Medical

**Published:** 1897-03-06

**Authors:** 


### NEW APPLIANCES AND THINGS MEDICAL.

[We shall be glad to receive, at our Office, 28 & 29, Southampton Street, Strand, London, W.C., from the manufacturers, specimens of all new preparations and appliances which may be brought out from time to time.]

### CYMRALIS.—A NEW TABLE WATER.

(R. Ellis and Son, Ruthin.)

This new table water has recently been added to the excellent list of mineral and aërated waters manufactured by R. Ellis and Son, of Ruthin. From time to time we have had occasion to call attention to the excellence of these waters, and after examination of "Cymralis" table water we have formed an opinion as favourable as that formed on previous occasions with respect to other samples submitted to us. It possesses all the best qualities that should be found in a good table water, for while its freedom from organic matter and general purity render it wholesome for drinking purposes, its highly aërated character makes it at the same time sparkling and refreshing. It lacks, moreover, that excess of saline constituents which render many of the popular varieties unfitted for combination with whiskey or other spirits. For the latter purpose Cymralis appears eminently fitted, and for medical and general use we believe this new water will still further enhance the reputation the Ruthin firm has already gained from the other waters and beverages it manufactures.

### APPARATUS FOR RAPID QUANTITATIVE ANALYSIS OF ALCOHOL, UREA, AND AMMONIA.

The clinical estimation, of alcohol and urea have been surrounded with so many difficulties that practically it has been found impossible for private individuals or physicians to undertake the responsibity; and, as a rule, it has been found expedient to submit the samples for analysis to some expert whose business it is to perform such analyses in a properly appointed laboratory. By the Barker-Smith thermometric method such analyses may now be undertaken with complete confidence by the veriest tyro. All that is necessary is a sample apparatus—the whole set costing six shillings—and a few simple solutions. The set consists of a thermometric experimental phial, tables, and pipette measure. The estimation of alcohol in spirits, tinctures, wines, &c., may be effected in about two minutes by the following method. Water is poured to the five-drachm mark in the phial, the temperature ascertained, and the wine or spirit poured to the second mark. The cork is replaced, the phial held by the rim, and the contents shaken for five seconds. The cork is then removed, and the temperature taken by the thermometric supplied. The rise in temperature is then ascertained, and the corresponding percentage of alcohol obtained from the printed table of percentages. The estimation of urea is equally simple, and is based on the rise of temperature caused by the addition of 1 c.c. of urine or fluid containing urea to 5 c.c. of chlorinated soda. Tables containing the percentage of urea corresponding to the rise in temperature are supplied with the apparatus. The estimation of ammonia is conducted in the same manner as above, but the tables supplied are of course different. The whole system is extraordinarily simple, and most ingenious. It is applicable, of course, only for uncomplicated estimations. Misleading factors may creep in and destroy the accuracy, but, practically, this is not likely to occur. We have tested the method, and compared it with other processes; and we are satisfied with its accuracy for clinical purposes. It should prove a real boon to the student and practitioner. The apparatus and particulars may be obtained from L. M. Smith, the Surgery, London, S.E.

### ASEPTIC MEMBRANE PERFORATOR.

We regret that in our notice of this last week the inventor's name was spelled Ribson, instead of Robson.

